# A diabetes update

**DOI:** 10.1111/1753-0407.13434

**Published:** 2023-06-13

**Authors:** Zachary Bloomgarden

**Affiliations:** ^1^ Department of Medicine Division of Endocrinology, Diabetes and Bone Disease, Icahn School of Medicine at Mount Sinai New York NY USA

Understanding of diabetes and its treatment is ever‐increasing. We summarize a variety of relevant studies – all potentially suggesting new approaches to the treatment of our patients.

## 1 BODY WEIGHT, OBESITY, AND GUT HORMONE‐BASED TREATMENT APPROACHES

Interrelationships between obesity and type 2 diabetes are the focus of a recent review, elegantly summarizing the effects of obesity‐related increase in diacyl glycerol and fatty acids on adipose tissue, on the liver, on skeletal muscle, and on the beta cell, with resistance to insulin action, abnormal vagal tone, and, perhaps, the gut microbiome as central players.^1^ In addition to obesity being associated both with diabetes and with cardiovascular disease (CVD), body weight variability is associated with development of diabetes, particularly among people with diabetes both with microvascular disease outcomes and, as shown in a recent review, with CVD outcomes. Such studies suggest that we should focus both on measures to reduce weight and to stabilize weight among people with diabetes, with glucagon‐like peptide (GLP)‐1 receptor activators and related medications offering a dual approach to both weight loss and weight stabilization.^2^

A review by Tschöp and colleagues of the various gut hormone combinations with potential for metabolic disease therapy illustrates current approaches with GLP‐1 plus glucose‐dependent insulinotropic polypeptide (GIP) receptor dual agonists such as tirzepatide (with high homology to exenatide), GLP‐1 plus glucagon receptor dual agonists such as cotadutide and SAR425899 and GLP‐1, GIP, and glucagon receptor triple agonists SAR441255 and LY3437943. In addition, GLP‐1 or glucagon receptor agonists have been linked to hormones acting at nuclear receptors for estrogen, thyroid hormone, and glucocorticoids, with intriguing evidence of potentiation of weight loss, and L‐cell secretagogues are being explored in combination with dipeptidyl peptidase‐4 inhibitors.^3^

An interesting post hoc study of 5378 participants with type 2 diabetes in phase 3 trials of tirzepatide analyzed the relationship found “modest (correlation coefficients 0.1438‐0.3130) association between HbA1c reduction and the effect on body weight,”^4^ suggesting that the two effects are at least partially independent. A study of the GLP‐1/GIP receptor activator tirzepatide administration in comparison to placebo in 2539 nondiabetic persons with obesity (mean body mass index 38) showed weight loss of 15%, 20%, and 21% versus 3% with 5 mg, 10 mg, and 15 mg weekly vs placebo, with the expected greater likelihoods of nausea, diarrhea, constipation, and dyspepsia, as well as alopecia in 5%–6% vs 1% with placebo, dizziness in 4%–6% vs 2% with placebo, severe depression in 0.3% versus none, gallbladder disease in 1%–2% vs 1%, hypoglycemia (glucose<54 mg/dL) in 1.5% vs none, cholecystitis in ~0.5% vs none, and pancreatitis in 0.2% of the treated vs placebo groups.^5^

An oral small molecule GLP‐1RA, danuglipron, was compared with placebo in 411 persons with type 2 diabetes, leading at the highest dose to placebo‐adjusted reduction in HbA1c by 1.16%, in fasting glucose by 33 mg/dL, and in body weight by 4.17 kg among completers, although with gastrointestinal symptoms of nausea and diarrhea, and with 14% of the participants withdrawing because of adverse events.^6^

## 2 BLOOD PRESSURE TREATMENT APPROACHES

A meta‐analysis of nine trials of 11 005 people with type 2 diabetes randomized to intensive blood pressure (BP) control (mean 125/73) vs conventional treatment (mean 134/79) showed 36% reduction in stroke and 32% reduction in progression to macroalbuminuria, although CVD and all‐cause mortality rates were similar at the two BP levels^7^; a similar meta‐analysis of six open‐label randomized controlled trials including 20 985 patients both having and not having diabetes showed reduction in CVD mortality and congestive heart failure as well as stroke.^8^ Studies of this sort imply that a BP target of <140/90 is insufficient for persons with diabetes having CVD; CVD risk factors; or Stage 3, 4, or 5 chronic kidney disease, for whom BP should be <130/80.^9^ Implementing such a target, however, must be tempered by the recognition that in‐office BP readings show high visit‐to‐visit variability and, as such, may not be appropriate in guiding management, leaving us with the dilemma of not having a practical gold standard to use in this crucial aspect of CVD management.^10^

## 3 INSULIN TREATMENT

Once‐weekly insulin icodec was compared with once‐daily insulin glargine U100 with 2–4 daily bolus insulin aspart injections in 582 persons with type 2 diabetes treated for 26 weeks, finding similar 1.2% reductions in HbA1c from 8.3% baseline, 32 vs 29 mg/dL reduction in fasting glucose, 2.7 vs 2.2 kg increases in body weight, and similar 37% vs 45% rates of level 1 hypoglycemia and 19% vs 25% rates of level 2 or 3 hypoglycemia.^11^ Reports of insulin icodec in type 1 diabetes^12^ and in insulin‐naïve type 2 diabetes^13^ also show improvement in glycemia similar to that with once daily insulin degludec, suggesting that it may soon be possible to use once weekly gut hormone‐based treatment and once weekly insulin, an approach likely to greatly improve the acceptability of such approaches to our patients.

It has been suggested that carbohydrate‐counting methods to estimate prandial insulin requirements for people with type 1 diabetes might not be necessary and that simplified qualitative meal‐size estimation might offer an easier approach to deciding on appropriate meal insulin doses.^14^ A study of 30 persons with type 1 diabetes using a closed‐loop insulin pump, however, compared two 3‐week periods and showed that when the participants crossed over between the two approaches their “time in range” of 70–180 mg/dL was 74.1% with carbohydrate counting, significantly greater than 70.5% with the qualitative method.^14^

## 4 DIABETIC RETINOPATHY TREATMENT

Fenofibrate may have benefit in diabetic retinopathy,^15^, ^16^ with a postulated mechanism involving blockade of hypoxia‐inducible factor (HIF)‐1.^17^ HIF‐1 acts in part by stimulating vascular endothelial growth factor (VEGF) release. In a recent report, a novel small molecule HIF inhibitor was shown to potently inhibit HIF‐regulated expression of VEGF and other vasoactive mediators thought to be involved in retinopathy pathogenesis, offering a new potential approach to retinopathy treatment.^18^ This may particularly be important in view of questions recently raised by a study suggesting that anti‐VEGF treatment of >14 000 persons with diabetic retinopathy was associated with increase in likelihood of myocardial infarction or overall cardiovascular disease,^19^ although the association may be confounded by the relationship between CVD and more severe degrees of retinopathy.^20^

## CONFLICT OF INTEREST STATEMENT

None.
